# Prevalence and age-related trends of refractive errors in Mexican outpatients: a cross-sectional study

**DOI:** 10.3389/fpubh.2025.1675138

**Published:** 2025-10-23

**Authors:** Abraham García-Gil, Beatriz Itzel Hernández-Jurado, Marco Antonio Luna-Ruiz-Esparza, Eduardo Espinoza-Angulo, Héctor Machado-Jiménez, Humberto Gómez-Campaña, Abraham Campos-Romero, Jonathan Alcántar-Fernández

**Affiliations:** ^1^Innovation and Research Department, Salud Digna, Culiacán, Mexico; ^2^Optometry Department, Salud Digna, Culiacán, Mexico; ^3^Medical Direction, Salud Digna, Culiacán, Mexico

**Keywords:** refractive errors, prevalence, astigmatism, myopia, hyperopia

## Abstract

**Background:**

Refractive errors (RE) are the leading cause of visual impairment and blindness worldwide. With ongoing demographic shifts, including population growth, aging, and lifestyle changes, the global prevalence of RE is expected to rise. Robust epidemiological data are essential to mitigate their public health impact. However, in Mexico, comprehensive studies assessing the burden of RE across age groups and regions are scarce.

**Objective:**

This study aimed to characterize the epidemiological profile of RE in Mexico using a large outpatient sample, focusing on geographic distribution and variation by sex and age.

**Methods:**

We analyzed an electronic database of eye examinations from 3.8 million outpatients who attended Salud Digna clinics across Mexico in 2024. Prevalence estimates were calculated with 95% confidence intervals (CIs), and statistical comparisons were performed to assess differences by sex and age.

**Results:**

The overall prevalence of RE was 74.61% (95% CI, 74.57–74.65) with 2,841,067 outpatients affected. Myopia was the most common condition, affecting 1,469,377 outpatients (38.6, 95% CI, 38.54–38.64), followed by hyperopia (1,234,403 affected outpatients, 32.4, 95% CI, 32.37–32.47) and astigmatism (1,205,400 affected outpatients, 31.7, 95% CI, 31.61–31.71). Astigmatism was significantly more prevalent in males across all age groups (*p* < 0.001). Myopia and hyperopia showed similar prevalence between sexes during childhood and early adulthood, but diverged in later adulthood, with higher rates of myopia in males (*p* < 0.001) and hyperopia in females (*p* < 0.001). All three RE types exhibited a marked shift in prevalence in the 40–49 age group. Geographic variation was observed, with central states showing higher astigmatism and myopia prevalences rates whereas coastal states showed higher hyperopia prevalence rates. Notably, a large proportion of outpatients have never undergone an eye examination, indicating substantial gaps in access to care.

**Conclusion:**

This outpatient-based study provides critical insights into the epidemiology of RE in Mexico, revealing significant demographic and geographic disparities. The findings underscore the urgent need for targeted public health strategies to improve access to eye care services, particularly for underserved populations and high-risk age groups.

## 1 Introduction

Vision impairment arises when an ocular condition impacts the visual system and one or more visual functions (1). It is estimated that at least 2.2 billion individuals globally experience vision impairment, and at least 1 billion cases could have been prevented. Vision impairment predominantly affects individuals in low- and middle-income countries and is more common among older populations (1, 2). The increasing population, aging, lifestyle changes, and urbanization are expected to significantly increase the proportion of individuals living with ocular conditions, visual impairment, and blindness in the coming decades, posing substantial challenges to public health systems (1).

Uncorrected refractive errors are the leading cause of vision impairment, followed by cataracts, age-related macular degeneration, glaucoma, and diabetic retinopathy (3, 4). Refractive errors (RE) are ocular conditions resulting from discrepancies between the eye’s axial length and its dioptric power, leading to blurry vision (5). There are three types of RE: myopia, characterized by excessive dioptric power relative to the eye’s axial length, causing blurry vision of distant objects (nearsightedness) (6), hyperopia, where insufficient dioptric power relative to the eye’s axial length results in blurry vision of nearby objects (farsightedness) (7); and astigmatism, where an irregular corneal surface causes distorted vision of both near and distant objects (7).

The most common symptoms of RE include blurry vision, diplopia, headaches, and asthenopia (8, 9). Given that some of these symptoms may go unnoticed, regular eye examinations are crucial for maintaining optimal visual health. RE is diagnosed through eye examination conducted by vision health professionals. Although RE cannot be cured, timely detection and professional treatment can mitigate vision impairment through corrective measures such as eyeglasses, contact lenses, or ocular surgery (8, 10). Despite the high cost-effectiveness of eyeglasses as a health intervention, it is estimated that globally, only 36% of individuals with RE have access to appropriate eyeglasses (11).

Meta-analyses have estimated that globally, the prevalence of astigmatism in children (14.9%) exceeds that of myopia (11.7%) and hyperopia (4.6%), with a similar pattern observed in adults: astigmatism (40.1%), myopia (26.5%), and hyperopia (30.9%). However, prevalence rates vary significantly between countries (12). Notably, a global analysis of RE prevalence indicated a sustained increase in myopia over time, increasing from 10.4% in 1993 to 34.2% by 2016. East Asia is the most affected region, with an estimated prevalence of 47% among adults aged 20 to 29 years (12). Projections estimate that by 2050, more than 50% of the global population will be affected by myopia (13).

In Latin America, the prevalence of visual impairment associated with UREs is estimated at 1.4% with 7.2 million people affected (14, 15). In Ecuador, UREs contributed to almost 30% of the total cases of visual impairment while contributing to 1.4% of blindness in Colombia, 2.1% in Chile, 2.3% in Brazil, and 5.0% in Venezuela (16).

Significant disparities in the prevalence of RE across different countries, ethnic groups, and age ranges indicate that both age and genetic factors contribute to its development. It has been implicated that genes involved in light response, light processing, and post-transcriptional regulation mediate a retina-to-sclera signaling cascade that may induce a scleral remodeling in response to light stimuli, influencing the onset of RE development (17, 18), which correlates with environmental factors, such as reduced exposure to natural light due to limited outdoor activity and increased near work, which have also been independently associated with the development of RE (5, 12).

Furthermore, outdoor activities have become increasingly limited. At the same time, digital screen use dominates both occupational and recreational time, leading not only to eye strain but also to musculoskeletal complaints, including neck, shoulder, and back pain (19). Nevertheless, as stated by Rosenfield et al. (19) “Perhaps it is not the form of the visual stimulus that matters, but rather factors such as working distance, gaze angle, and the length of time spent viewing the display without breaks.” Beyond vision impairment, RE have a substantial social and economic impact, limiting employment and educational opportunities, and diminishing productivity (1).

The 2020 Population and Housing Census in Mexico reported that 2,691,338 individuals have a visual impairment (20). Additional studies estimate that 11.01 million Mexicans (8.7% of the population) are either blind or have a visual impairment, with RE accounting for 2.61 million cases (21). Unfortunately, Mexico lacks a national registry for individuals with visual health conditions, and existing research has predominantly focused on school-aged children. There is a notable deficiency of nationally representative studies encompassing all age groups and all three types of refractive errors (RE) in relation to the burden of RE in Mexico. This absence of comprehensive, population-based research that includes all age groups and RE types hinders the development of targeted public health strategies.

This study addresses a critical gap by analyzing the largest outpatient dataset of refractive errors (RE) in Mexico to date, collected from Salud Digna, a non-profit institution with over 200 clinics nationwide and the leading provider of eyeglasses in the country since 2015. REs are the leading cause of vision impairment globally, yet they are among the most easily preventable and treatable ocular conditions. Uncorrected REs can progress to avoidable blindness, particularly in underserved populations, making their detection and correction a key priority for global eye health.

Using data from patients examined in 2024, we present a comprehensive analysis of RE prevalence stratified by age, sex, and geographic region. We highlight age-related trends, including a notable inflection point in the 40–49 age group, and report both relative and absolute numbers of affected individuals. These findings aim to inform public health strategies, support screening and treatment programs, and directly contribute to the WHO’s VISION 2030 initiative, which seeks to reduce avoidable blindness and ensure universal access to eye care services (1, 22).

## 2 Methods

### 2.1 Study population

In a cross-sectional, retrospective study, we examined anonymized electronic health records from non-cycloplegic eye exams of all eligible outpatients who visited Salud Digna clinics across all 32 Mexican states between 1 January 2024 and 31 December 2024.

Our clinics follow a standardized eye examination procedure, always conducted by a certified optometrist. The process begins with verifying the patient’s identity and completing a clinical history questionnaire. All equipment is meticulously sanitized. If the patient wears corrective lenses, a lensometry test is performed. The patient’s interpupillary and nasopupillary distances are measured, and their uncorrected visual acuity is assessed. A refractometer (AKR550, WAM 800, Essilor Instruments, Paris, France) is used to evaluate the individual’s objective refraction. The optometrist then refines the objective prescription and assesses both distance and near visual acuity. The results of the eye exam, including any refractive errors and recommendations for eyeglass materials and usage, are shared with the patient. If necessary, the patient is referred to an ophthalmologist or a retinography service.

Data collected during eye examinations, including demographic information, were included. A unique, standardized, validated survey was used to collect data from all clinics. Data from the right eye were used throughout the whole study, and RE were defined as follows: myopia, ≤−0.50 diopter (D) spherical equivalent (SE) (6); hyperopia, ≥0.50 D SE (7); astigmatism, ≤ −1.00 D cylinder (7).

### 2.2 Consent for using information, handling data, and protecting information privacy

Consent for using information from health records was obtained in accordance with the Mexican Federal Law on Personal Data Protection (LFPDPPP, Spanish acronym). Individuals attending Salud Digna clinics agreed to our privacy policy, which includes the use of their information for scientific research purposes. Therefore, specific informed consent from each individual included in this study was not required, as this research involved a cross-sectional analysis of an electronic health registry. Data protection and privacy were managed in accordance with the national laws and guidelines of Mexico. The data were anonymized by assigning a unique ID code to protect the individual’s identity and prevent duplication in subsequent analyses.

### 2.3 Ethical statement

This study was approved by the Ethical Review and Research Board of Salud Digna (SDI-202503). All methods adhered to the approved guidelines for clinical management information, the Declaration of Helsinki, and the national regulations of the country (Federal Law on Data Privacy Protection). The data were anonymized for the study.

### 2.4 Statistical analysis

Descriptive statistical analyses were conducted using Microsoft Power Query and Microsoft Excel to summarize demographic and clinical characteristics of the study population. All inferential statistical analyses were performed in R (version 4.4.0) (23). Prevalence estimates and age-adjusted prevalence rates of RE were calculated using the dplyr package (v1.1.4) and the ageadjust.direct() function from the epitools package (v0.5–10.1). Age standardization was performed using the direct method, applying the World Health Organization (WHO) standard population as the reference to minimize age-related bias across Mexican states and between sexes (24).

Exact 95% confidence intervals (CIs) for prevalence proportions were computed using the binom.exact() function from the epitools package, which applies the Clopper-Pearson method based on the binomial distribution. This method was chosen for its accuracy in estimating CIs for proportions.

Chi-square tests for independence were conducted using the chisq.test() function from the base stats package to assess associations between categorical variables. Risk ratios (RRs) and their corresponding 95% CIs were estimated using the riskratio() function from the epitools package, which calculates the ratio of probabilities of the outcome occurring in exposed versus unexposed groups.

To evaluate trends in age-specific prevalence, we applied the chi-squared test for trend using the prop.trend.test() function from the stats package. This test assesses whether there is a statistically significant linear trend in proportions across ordered age groups.

All statistical tests were two-sided, and a *p*-value < 0.05 was considered statistically significant.

## 3 Results

Between 1 January 2024 and 31 December 2024, 3,807,547 individuals underwent eye examinations at Salud Digna diagnostic clinics. The mean age of the individuals was 42.16 years (SD 19.7); of these, 2,382,045 were female (62.6%) and 1,425,502 were male (37.4%). Among the participants, 1,872,681 (49.2%) declared never having undergone a previous eye examination, and 3,733,240 (98%) indicated that they had not undergone an ophthalmologic evaluation. The most common symptom was blurry vision of distant objects, reported by 2,329,589 individuals (61.2%), followed by blurry vision of near objects reported by 1,879,567 (49.4%), while 679,827 (17.9%) reported no symptoms. Additionally, 366,087 and 471,602 (9.6 and 12.4%, respectively) of the overall individuals declared having a previous diabetes or high blood pressure diagnosis. Table 1 summarizes the characteristics of the individuals included in this study.

**Table 1:** Demographic characteristics of the outpatients included in this study.

Characteristic	*n*	%
Sex		
Female	2,382,045	62.6%
Male	1,425,502	37.4%
Age	42.16 ± 19.7	
<10	108,986	2.9%
10–19	539,248	14.2%
20–29	566,093	14.9%
30–39	431,070	11.3%
40–49	623,186	16.4%
50–59	725,828	19.1%
60–69	512,225	13.5%
70–79	236,878	6.2%
≥80	64,033	1.7%
Previous eye examination		
Yes	1,887,759	49.6%
No	1,872,681	49.2%
Missed	47,107	1.2%
Previous ophthalmologic revision		
Yes	56,730	1.5%
No	3,733,240	98.0%
Missed	17,577	0.5%
Use of glasses		
Yes	1,983,451	52.1%
No	1,776,989	46.7%
Missed	47,107	1.2%
Symptoms		
Blurred far vision	2,329,589	61.2%
Blurred near vision	1,879,567	49.4%
Headache	121,474	3.2%
Eye pain	76,123	2.0%
Itchy eyes	30,739	0.8%
None	679,827	17.9%
Diabetes		
Yes	366,087	9.6%
No	3,424,308	89.9%
Missed	17,152	0.5%
High blood pressure		
Yes	471,602	12.4%
No	3,315,287	87.1%
Missed	20,658	0.5%

**Figure 1:**
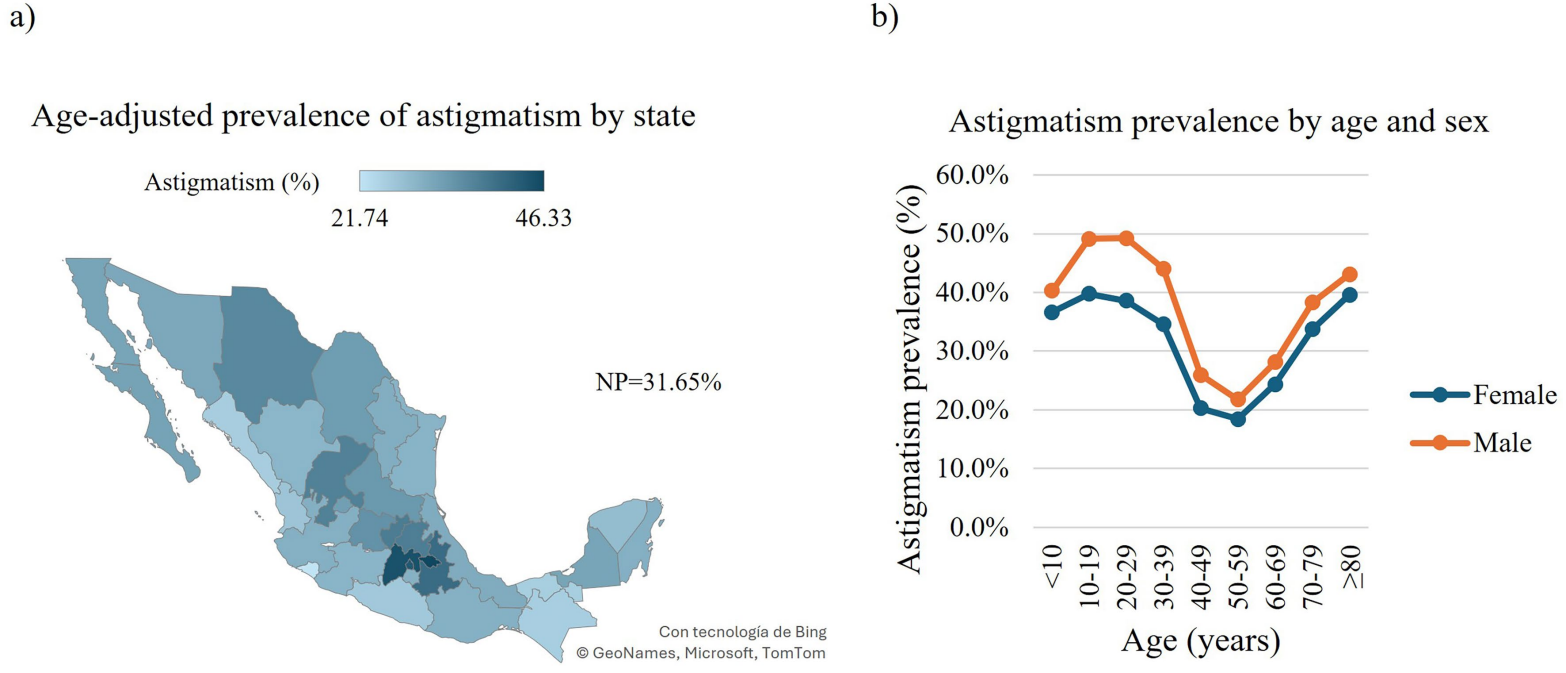
Prevalence of astigmatism in Mexican outpatients. Choropleth map of the age-adjusted prevalence of astigmatism in Mexican outpatients by state **(a)**. Prevalence of astigmatism in Mexican outpatients by age and sex **(b)**. Trend of age-specific prevalence evaluated with the chi-squared (*χ*2) test for trend: Females: *χ*2 = 32,353, df = 1, *p*-value < 0.0001; males: *χ*2 = 34,451, df = 1, *p*-value < 0.0001. NP, national prevalence.

**Figure 2:**
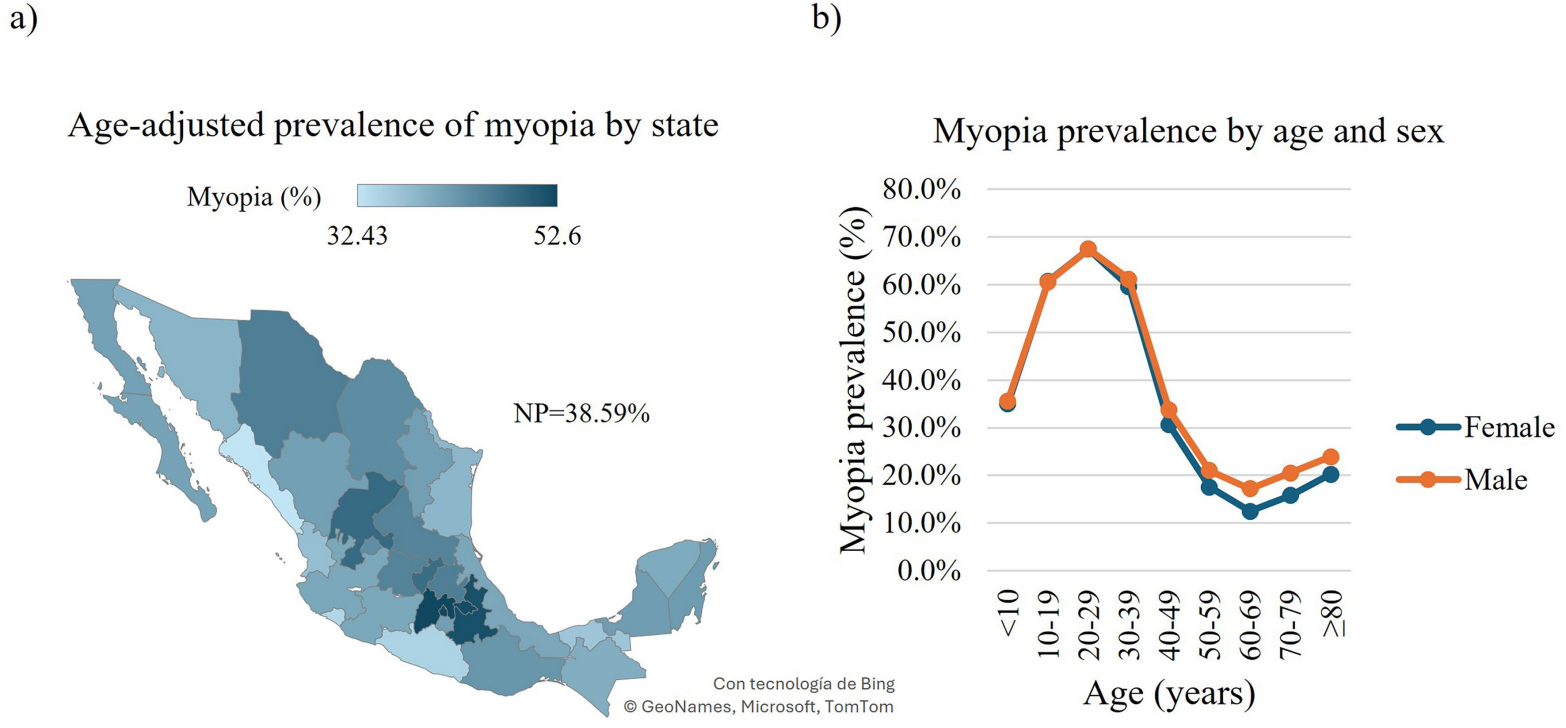
Prevalence of myopia in Mexican outpatients. Choropleth map of the age-adjusted prevalence of myopia in Mexican outpatients by state **(a)**. Prevalence of myopia in Mexican outpatients by age and sex **(b)**. Trend of age-specific prevalence evaluated with the chi-squared (*χ*2) test for trend: Females: *χ*2 = 353,458, df = 1, *p*-value < 0.0001; males, *χ*2 = 161,030, df = 1, *p*-value < 0.0001. NP, national prevalence.

**Figure 3:**
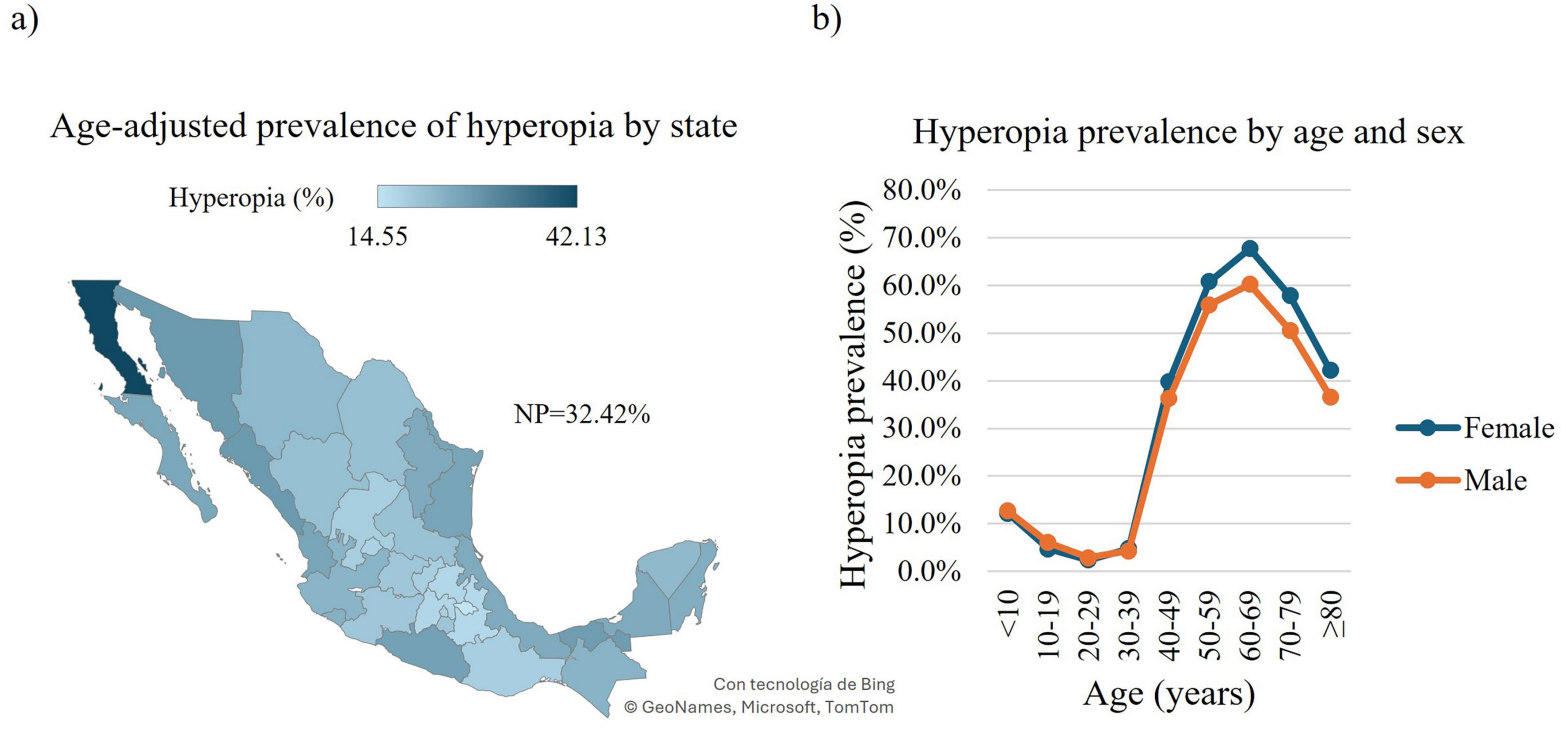
Prevalence of hyperopia in Mexican outpatients. Choropleth map of the age-adjusted prevalence of hyperopia in Mexican outpatients by state **(a)**. Prevalence of hyperopia in Mexican outpatients by age and sex **(b)**. Trend of age-specific prevalence evaluated with the chi-squared (*χ*2) test for trend: Females, *χ*2 = 110,443, df = 1, *p*-value < 0.0001; males, *χ*2 = 71,020, df = 1, *p*-value < 0.0001. NP = national prevalence.

Concerning the overall RE, we found 2,841,067 affected individuals (74.61, 95% CI: 74.57–74.65), with myopia being the most prevalent affecting 1,469,377 outpatients (38.6, 95% CI: 38.54–38.64), followed by hyperopia and astigmatism affecting 1,234,403 (32.4, 95% CI: 32.37–32.47) and 1,205,400 (31.7, 95% CI: 31.61–31.71) outpatients, respectively (Figures 1A, 2A, 3A, Supplementary Table 1). When stratified by compound RE, the prevalence rates were as follows: simple astigmatism, 3.6% (137,238 outpatients, 95% CI: 3.58–3.62); myopic astigmatism, 22.2% (846,16 outpatients, 95% CI:22.18–22.26); hyperopic astigmatism, 5.8% (222,034 outpatients, 95% CI:5.80–5.85); simple myopia, 16.4% (623,234 outpatients, 95% CI:16.30–16.40); and simple hyperopia, 26.6% (1,012,395 outpatients, 95% CI:26.54–26.63) (Supplementary Table 1).

Regarding the national distribution of astigmatism, Tlaxcala exhibited the highest age-adjusted prevalence affecting 5,147 outpatients (46.3, 95% CI: 44.61–48.13), followed by Estado de Mexico with 235,387 affected outpatients (44.8, 95% CI: 44.58–45.03). In contrast, Colima and Chiapas showed the lowest age-adjusted prevalence with 6,284 (21.7, 95% CI: 20.98–22.53) and 16,787 (25.2, 95% CI: 24.57–25.77) affected outpatients, respectively (Figure 1A, Supplementary Table 2). When analyzed by sex, males presented a higher age-adjusted overall prevalence of astigmatism with 516,154 (39.3, 95% CI: 39.17–39.44) affected outpatients than females with 689,246 affected outpatients (32.4, 95% CI: 32.32–32.54) (Supplementary Table 3). We observed comparable patterns in the prevalence of astigmatism across sexes with regard to age groups. In both males and females, the prevalence increased with age, peaking during the 10–29-year age range (females: 10–19 years, 125,497 outpatients, 39.7 95% CI: 39.6–39.9; 20–29 years, 137,372 outpatients, 38.6 95% CI: 38.5–38.8; males: 10–19 years, 109,822 outpatients, 49.1 95% CI: 48.9–49.3; 20–29 years, 103,576 outpatients, 49.2 95% CI: 49.0–49.4). This was followed by a declining trend, reaching the lowest prevalence in the 50–59-year age group (females: 86,456 outpatients, 18.4 95% CI: 18.3–18.5; males: 55,793 outpatients, 21.7 95% CI: 21.6–21.9). Thereafter, the prevalence began to rise again, closely returning to the peak levels observed in early adulthood. (Figure 1B).

Concerning the national distribution of myopia, Estado de Mexico exhibited the highest age-adjusted prevalence with 280,488 affected outpatients at 52.6% (95% CI: 52.37–52.84), followed by Ciudad de Mexico with 193,529 affected outpatients at 52.5% (95% CI: 52.24–52.82). In contrast, Sinaloa and Colima showed the lowest age-adjusted prevalence with 50,497 (32.4, 95% CI: 32.08–32.78) and 8,394 (34.4, 95% CI: 33.45–35.37) affected outpatients, respectively (Figure 2A, Supplementary Table 2). When analyzed by sex, males presented a higher age-adjusted overall prevalence of myopia with 577,936 affected outpatients (45.1, 95% CI: 45.01–45.28) than females with 891,441 affected outpatients (43.5, 95% CI: 43.42–43.66; Supplementary Table 3). We identified similar age-related patterns in the prevalence of myopia among males and females. In both sexes, the prevalence increased markedly and consistently, reaching a peak in the 20–29-year age group (females: 239,978 affected outpatients 67.5 95% CI: 67.3–67.6; males: 141,867 affected outpatients, 67.4 95% CI: 67.2–67.6). This was followed by a declining trend, with the lowest prevalence observed in the 60–69-year age group (females: 40,165 affected outpatients, 12.5 95% CI: 12.3–12.6; males: 32,627 affected outpatients, 17.2 95% CI: 17.0–17.3). Subsequently, prevalence began to rise again, although it did not return to the peak levels observed in early adulthood (Figure 2B).

Regarding the national distribution of hyperopia, Baja California exhibited the highest age-adjusted prevalence with 88,217 affected outpatients at 42.1% (95% CI: 41.79–42.49), followed by Sonora with 59,901 affected outpatients at 27.8% (95% CI: 27.5–28.08). In contrast, Tlaxcala and Puebla showed the lowest age-adjusted prevalence with 2,279 (14.5, 95% CI: 13.68–15.52) and 36,993 (16.6, 95% CI: 16.36–16.82) affected outpatients, respectively (Figure 3A, Supplementary Table 2). When analyzed by sex, females presented a higher age-adjusted overall prevalence of hyperopia with 809,299 affected outpatients (22.4, 95% CI: 22.34–22.48) than males with 425,104 affected outpatients (20.1, 95% CI: 20.87–21.03; Supplementary Table 3). We observed comparable age-related trends in the prevalence of hyperopia among men and women. In both sexes, the prevalence declined with age, reaching its lowest point in the 20–29-year age group (females: 8,360 affected outpatients, 2.4 95% CI: 2.3–2.4; males: 6,029 affected outpatients 2.9 95% CI: 2.8–2.9). This was followed by a steady increase, peaking in the 60–69-year age group (females: 218,365 affected outpatients 67.8% CI: 67.6–67.9; males: 114,473 affected outpatients, 60.3 95% CI: 60.1–60.5). The prevalence began to decline again, although it did not return to the minimum levels observed in early adulthood (Figure 3B).

## 4 Discussion

As previously noted, demographic growth, population aging, lifestyle changes, and increasing urbanization are projected to substantially elevate the global burden of ocular conditions. Comprehensive epidemiological research is essential to effectively mitigate the impact of RE and other vision-related disorders. Such data can inform the development of targeted public health strategies at both the local and national levels, ultimately contributing to a reduction in the prevalence and impact of these conditions.

Unfortunately, Mexico lacks comprehensive national studies that encompass all 32 states, all age groups, and all three types of refractive errors (RE). This research constitutes the most extensive examination of refractive errors in Mexico to date, utilizing a database that includes outpatients from every age group and region across the nation. Its extensive national reach and large sample size provide a detailed and representative overview of the visual health of the Mexican population, rendering it a crucial resource for public health decision-making. The study involved a significant number of outpatients and covered the entire Mexican territory. By analyzing clinical eye examination data from Salud Digna, we aimed to map the national distribution of RE and explore its variations by gender and age. It is important to consider that our findings are based on an outpatient population. Nonetheless, the results are intended to aid vision health professionals and policymakers in crafting strategies to enhance access to eye care services and to guide policies on screening, treatment, and long-term management of RE in the future.

### 4.1 National astigmatism prevalence

In examining the prevalence of astigmatism, our findings indicate a higher prevalence than the global estimates for children as reported by Hashemi et al. (12), yet lower than the figures reported for adults in their study. Within the context of Mexican research, our observed prevalence of astigmatism surpasses that documented in most previous studies (25–30), with the exception of Barba-Gallardo et al. (31), who reported a higher prevalence than that identified in our study. The observed discrepancies in estimates not only for astigmatism, but also for myopia, and hyperopia can be attributed to differences in sample collection, sample size, the definition of refractive error (RE) employed, as well as genetic and ethnic factors, complicating direct comparisons of RE prevalence between this study and prior research. Regarding the geographical distribution of astigmatism cases, a higher prevalence is noted in the central region of the country, which is both highly industrialized and densely populated. Genetic factors, given Mexico’s status as one of the most ancestrally diverse nations in the Americas (32), alongside environmental factors, may contribute to these observed patterns.

### 4.2 National myopia prevalence

In recent decades, there has been a concerning global increase in the prevalence of myopia, particularly in Asian countries such as China, South Korea, and Singapore (13). This trend has been attributed to factors including rapid urbanization, intensified educational activities, prolonged use of electronic devices, and reduced outdoor time. These developments raise public health concerns due to the risk of high myopia and its associated visual complications (13).

The prevalence of myopia in our study exceeded the global figures reported by Hashemi et al. (12) and Holden et al. (13), although it remained lower than the prevalence recently reported by Hönekopp et al. (33) among German primary school children. In the context of myopia estimates in Mexico, our findings indicate a higher prevalence than that documented by previous authors (25–31). As with astigmatism, these differences in estimates can be attributed to variations in distinct factors as previously stated.

In line with the geographical distribution of astigmatism cases, myopia was found to be more common in the central part of the country. This is expected, as many of the myopia cases identified in this study also involve astigmatism. Genetic factors might play a role in these patterns, along with environmental influences such as the balance between near and distance vision tasks, since outdoor activities have been shown to significantly delay both the onset and progression of myopia (34). In Mexico, factors like rapid urbanization, an aging population, increased screen time, and decreased outdoor activities may be contributing to the rise in myopia and other refractive errors. Recent research has connected these factors to changes in visual behavior, particularly among children and adolescents, highlighting the importance of considering these elements in preventive measures (35, 36).

### 4.3 National hyperopia prevalence

Our study identified a higher prevalence of hyperopia compared to the global figures reported by Hashemi et al. (12) and Delpizzo et al. (37). Conversely, when compared to Mexican studies, the prevalence of hyperopia in this study aligns with the national estimates provided by Ramirez-Ortiz et al. (26) for primary school children. This prevalence exceeds the figures reported by Ortiz et al. (25) for the general population, slightly surpasses those reported by Teran et al. (27, 28, 30) for school-aged children (6–18 years), and is marginally below the prevalence noted by Barba-Gallardo et al. (31) in children. However, it remains higher than the prevalence reported by Barba-Gallardo et al. (31) in adults.

Similar to astigmatism, the differences in estimates can be attributed to variations in several factors as previously mentioned.

The analysis of the geographical distribution of refractive errors (RE) indicated a higher prevalence of hyperopia in coastal states. The reasons behind the coastal distribution of hyperopia cases warrant further investigation. As with the geographical distribution of astigmatism and myopia, genetics may play a role, although there is significant genetic variation in the Mexican coastal states exhibiting these patterns (32). Therefore, environmental factors, such as the frequency of near versus distance vision tasks, might be more influential, as the Mexican coastal states are less industrialized and densely populated compared to the central region.

It is important to note that our study encompasses all 32 Mexican states and includes participants from all age groups. In contrast, previous studies on the prevalence of RE in Mexico have primarily been regional in scope, with specific areas underrepresented (25), or have predominantly focused on specific age groups, particularly school-aged children. These differences, along with substantial variations in sample size, may partially account for the discrepancies between our findings and those of earlier reports. Additionally, it is worth noting that our study population consists of self-selected outpatients seeking optical services. In contrast, some previous studies have employed population-based screening approaches targeting the general public which may lead to an overestimation in our study.

### 4.4 RE relation to sex and age

Our investigation into the association between refractive error (RE), gender, and age revealed that males consistently exhibited a higher prevalence of astigmatism compared to females across all age categories. In contrast, the incidence of myopia and hyperopia was comparable between the sexes during childhood and early adulthood. However, as outpatients aged, a divergence emerged, with males demonstrating a greater propensity for myopia and females for hyperopia. The gender-based differences in refractive error prevalence may be attributed to hormonal, occupational, and behavioral factors. For instance, sex hormones may affect ocular biomechanics, while variations in occupational roles and visual habits between males and females could account for the increased incidence of myopia in males and hyperopia in females in older age groups (38–40).

Our findings also suggest a subtle physiological transition in the visual system beginning in the 30–39 age range, which becomes more pronounced in the 40–49 age group, where a marked shift in the prevalence of all three types of RE is observed. This pattern may be linked to age-related biomechanical changes in the ocular structures. Notably, previous research has identified two distinct aging-related metabolomic shifts occurring around the ages of 44 and 60 (41), which could underline the changes in RE prevalence in the population of the current study. However, the specific factors and their interactions that contribute to the observed sex differences, with males exhibiting a greater tendency toward myopia and females toward hyperopia in these age groups, remain poorly understood and require further research.

To further explore sex as a potential risk factor for RE development, we calculated risk ratios (Supplementary Table 5). Although these analyses yielded statistically significant results, they should be interpreted cautiously. The large sample size in our study increased the likelihood of detecting statistically significant differences, which may not necessarily reflect clinically meaningful effects. Similarly, chi-square tests assessing sex-based differences in RE prevalence produced several statistically significant outcomes (Supplementary Table 6); however, many of these may be spurious and should also be interpreted within a clinical, rather than purely statistical, context.

Our research indicates that myopia and astigmatism are highly prevalent among children and young adults, presenting significant social and economic challenges by limiting educational achievements, employment opportunities, and overall productivity (1). Uncorrected refractive errors constitute a major cause of preventable visual impairment, adversely affecting academic performance, work efficiency, and quality of life. Furthermore, the cost of optical correction can be substantial, particularly for at-risk populations (15, 42). Therefore, it is imperative to implement visual screening initiatives and public policies that ensure access to affordable eyewear and strategies to mitigate myopia progression in children.

In contrast, hyperopia was more prevalent in older adults, representing a substantial burden in this population group. This is particularly noteworthy, as visual impairment in later life is strongly associated with reduced quality of life and increased risk of falls and fractures (43).

### 4.5 Strengths and limitations

The strength of our study lies in its large, nationally representative sample, encompassing all 32 states of Mexico, making our findings highly generalizable and reflective of the Mexican population. Additionally, the eye examination procedures are standardized across our clinics and are consistently performed by certified optometrists.

It is important to highlight that the data used in this research were derived from eye examinations conducted without cycloplegia. Differences between refractive evaluations with and without cycloplegia have been reported, as non-cycloplegic assessments might overestimate myopia and underestimate hyperopia, especially in younger individuals (44, 45). This variation could potentially lead to bias when determining the prevalence of these refractive errors in children and young adults.

Additionally, the age recorded in our study corresponds to the time of the eye examination, which may not reflect the true age at which the refractive error developed. This could introduce a degree of reporting bias in the age-related prevalence estimates.

Although data on systemic conditions such as hypertension and diabetes were collected, they were not analyzed as stratifying variables due to the primary focus of the study. Future research may explore these associations in greater depth.

Our study group comprised outpatients who independently opted to seek diagnostic services at Salud Digna, resulting in a self-selected cohort that included both insured and uninsured participants from diverse socioeconomic backgrounds. This self-selection may introduce potential bias, as these outpatients might already exhibit symptoms warranting an eye examination, potentially leading to an overestimation of the prevalence of refractive errors compared to the general population. Although this self-selection presents a possible bias, it is somewhat mitigated by the extensive data collected, which allows for more detailed analyses under specific conditions of interest. Therefore, the results derived from the Salud Digna data should be interpreted with an understanding of this population, taking into account the inherent selection bias.

### 4.6 Concluding remarks

Reliable data on RE in Mexico remains scarce, limiting the development of effective public health strategies. This study provides a comprehensive overview of the current landscape of RE in the country, aiming to promote multisectoral collaboration both within Mexico and in nations with similar cultural and ancestral contexts.

One of the most pressing public health findings is the substantial proportion of outpatients who have never undergone an eye examination, revealing critical gaps in access to basic eye care services. Furthermore, the high unmet need for corrective lenses highlights the urgency of implementing policies that improve the availability, affordability, and accessibility of vision screening and eyeglasses.

Addressing these challenges requires coordinated efforts between public and private sectors. Strategic partnerships can empower communities by reallocating infrastructure and human resources to strengthen ophthalmological services and preventive care. These initiatives should prioritize early detection and treatment of RE and other visual conditions, especially among the most affected groups identified in this and other studies, namely, school-aged children and adults over 40 years of age.

To advance visual health equity, it is essential to integrate eye care into national public health agendas. Longitudinal studies and population-based surveys using standardized methodologies are recommended to monitor the evolution of RE, assess the impact of interventions, identify emerging risk factors, and guide future policy decisions.

## Data Availability

The datasets presented in this article are not readily available because under the Mexican Federal Law on Personal Data Protection we are not allowed to. Requests to access the datasets should be directed to jonathan.alcantar@salud-digna.org.

## References

[1] WHO. World report on vision, vol. 214. Geneva: World health Organisation (2019).

[2] Dai M Ouyang Y. Global, regional, and national burden of refraction disorders: findings from the global burden of disease study 2021 and projections to 2050. BMC Public Health. (2025) 25:1247. doi: 10.1186/s12889-025-22440-w, PMID: 40175971 PMC11966897

[3] Bourne RRA Steinmetz JD Saylan M Mersha AM Weldemariam AH Wondmeneh TG. Causes of blindness and vision impairment in 2020 and trends over 30 years, and prevalence of avoidable blindness in relation to VISION 2020: the right to sight: An analysis for the global burden of disease study. Lancet Glob Health. (2021) 9:e144–60. doi: 10.1016/S2214-109X(20)30489-7 33275949 PMC7820391

[4] Fricke TR Tahhan N Resnikoff S Papas E Burnett A Ho SM. Global prevalence of presbyopia and vision impairment from uncorrected presbyopia: systematic review, Meta-analysis, and modelling. Ophthalmology. (2018) 125:1492–9. doi: 10.1016/j.ophtha.2018.04.013, PMID: 29753495

[5] Harb EN Wildsoet CF. Origins of refractive errors: environmental and genetic factors. Annu Rev Vis Sci. (2019) 5:47–72. doi: 10.1146/annurev-vision-091718-015027 31525141 PMC11827892

[6] Baird PN Saw SM Lanca C Guggenheim JA Smith Iii EL Zhou X. Myopia (primer). Nat Rev Dis Primers. (2020) 6:99. doi: 10.1038/s41572-020-00231-4 33328468

[7] Galvis V Tello A Camacho PA Gómez LM Rey JJ Serrano AA. Definition of refractive errors for research studies: spherical equivalent could not be enough. J Optom. (2021) 14:224–5. doi: 10.1016/j.optom.2020.10.003, PMID: 33402285 PMC8093534

[8] National Eye Institute. Refractive errors (2023). Available online at: https://www.nei.nih.gov/learn-about-eye-health/eye-conditions-and-diseases/refractive-errors (Accessed 15, 2024).

[9] Kierstan Boyd. What is presbyopia? (2022), Am Acad Ophthalmol. Available online at: https://www.aao.org/eye-health/diseases/what-is-presbyopia (Accessed May 15, 2024).

[10] Jacobs DS Afshari NA Bishop RJ Keenan JD Lee J Shen TT. Refractive errors preferred practice pattern®. Ophthalmology. (2023) 130:P1–P60. doi: 10.1016/j.ophtha.2022.10.031 36543603

[11] WHO. Blindess and visual impairment (2023). Available online at: https://www.who.int/news-room/fact-sheets/detail/blindness-and-visual-impairment (Accessed May 15, 2024)

[12] Hashemi H Fotouhi A Yekta A Pakzad R Ostadimoghaddam H Khabazkhoob M. Global and regional estimates of prevalence of refractive errors: systematic review and meta-analysis. J Curr Ophthalmol. (2018) 30:3–22. doi: 10.1016/j.joco.2017.08.009 29564404 PMC5859285

[13] Holden BA Fricke TR Wilson DA Jong M Naidoo KS Sankaridurg P. Global prevalence of myopia and high myopia and temporal trends from 2000 through 2050. Ophthalmology. (2016) 123:1036–42. doi: 10.1016/j.ophtha.2016.01.006, PMID: 26875007

[14] Durr NJ Dave SR Lage E Marcos S Thorn F Lim D. From unseen to seen: tackling the global burden of uncorrected refractive errors. Annu Rev Biomed Eng. (2014) 16:131–53. doi: 10.1146/annurev-bioeng-071813-105216, PMID: 24905874

[15] Resnikoff S Pascolini D Mariotti SP Pokharel GP. Global magnitude of visual impairment caused by uncorrected refractive errors in 2004. Bull World Health Organ. (2008) 86:63–70. doi: 10.2471/BLT.07.041210, PMID: 18235892 PMC2647357

[16] Furtado JM Lansingh VC Carter MJ Milanese MF Peña BN Ghersi HA. Causes of blindness and visual impairment in Latin America. Surv Ophthalmol. (2012) 57:149–77. doi: 10.1016/j.survophthal.2011.07.002, PMID: 22137039

[17] Tedja MS Wojciechowski R Hysi PG Eriksson N Furlotte NA Verhoeven VJM. Genome-wide association meta-analysis highlights light-induced signaling as a driver for refractive error. Nat Genet. (2018) 50:834–48. doi: 10.1038/s41588-018-0127-7, PMID: 29808027 PMC5980758

[18] Hysi PG Choquet H Khawaja AP Wojciechowski R Tedja MS Yin J. Meta-analysis of 542,934 subjects of European ancestry identifies new genes and mechanisms predisposing to refractive error and myopia. Nat Genet. (2020) 52:401–7. doi: 10.1038/s41588-020-0599-0, PMID: 32231278 PMC7145443

[19] Rosenfield M Kollbaum P Prenat O. Eye strain and near work: both an ancient problem and a modern concern. Ophthalmic Physiol Opt. (2025) 45:1037–9. doi: 10.1111/opo.13520, PMID: 40299670

[20] INEGI. ESTADÍSTICAS A PROPÓSITO DEL DÍA INTERNACIONAL DE LAS PERSONAS CON DISCAPACIDAD (DATOS NACIONALES) (2021). Available online at: https://www.inegi.org.mx/contenidos/saladeprensa/aproposito/2021/EAP_PersDiscap21.pdf (Accessed May 20, 2024).

[21] Madueña-Angulo SE Beltran-Ontiveros SA Leal-Leon E Contreras-Gutierrez JA Lizarraga-Verdugo E Gutierrez-Arzapalo PY. National sex- and age-specific burden of blindness and vision impairment by cause in Mexico in 2019: a secondary analysis of the global burden of disease study 2019. Lancet Reg Health Am. (2023) 24:100552. doi: 10.1016/j.lana.2023.100552 37457139 PMC10339251

[22] World Health Organization. SPECS 2030. (2025). Available online at: https://www.who.int/initiatives/specs-2030 (Accessed September 16, 2025)

[23] R Core Team. R: a language and environment for statistical computing. Vienna, Austria: R Foundation for Statistical Computing (2023).

[24] Ahmad OB Boschi-Pinto C Lopez AD Murray CJ Lozano R Inoue M. Age standardization of rates: A new WHO standard. GPE Discussion Paper Series: No. 31. Geneva: World Health Organization (2001). 31 p.

[25] Ortiz MI Campuzano Revilla GP Muñoz Pérez V Cuevas Suárez CE. Prevalencia de miopía, hipermetropía y astigmatismo en México: Una revisión sistemática. Educación y Salud Boletín Científico Instituto de Ciencias de la Salud Universidad Autónoma del Estado de Hidalgo. (2022) 10. doi: 10.29057/icsa.v10i20.8591

[26] Ramírez-Ortiz MA Amato-Almanza M Romero-Bautista I Klunder-Klunder M Aguirre-Luna O Kuzhda I. A large-scale analysis of refractive errors in students attending public primary schools in Mexico. Sci Rep. (2023) 13:13509. doi: 10.1038/s41598-023-40810-5, PMID: 37598286 PMC10439951

[27] Teran E Ramírez-Jaime R Martínez-Gaytán C Romo-García E Costela FM. Refractive error of students (15- to 18-year-olds) in Northwest Mexico. Optom Vis Sci. (2021) 98:1127–31. doi: 10.1097/OPX.0000000000001779, PMID: 34629438

[28] Terán E Romo-García E Félix-Medina MH Martínez-Gaytán C Ramírez-Jaimes R Santiago H. Refractive error of students (12–15-years-old) in northwestern Mexico. Revista Mexicana de Oftalmología. (2023) 97:73–9. doi: 10.5005/rmo-11013-0043

[29] Gomez-Salazar F Campos-Romero A Gomez-Campaña H Cruz-Zamudio C Chaidez-Felix M Leon-Sicairos N. Refractive errors among children, adolescents and adults attending eye clinics in Mexico. Int J Ophthalmol. (2017) 10:796–802. doi: 10.18240/ijo.2017.05.23 28546940 PMC5437471

[30] Teran E Romo-García E Santiago HC. Refractive errors of school children from economically disadvantaged areas in Northwest México. J Clin Med. (2024) 13:3094. doi: 10.3390/jcm13113094, PMID: 38892805 PMC11172553

[31] Barba-Gallardo LF Salas-Hernández LH Villafán-Bernal JR Marín-Nájera P d S García-López DM López-Garcia ADC. Refractive status of patients attending eye clinics of the public health system from Aguascalientes, Mexico. J Optom. (2021) 14:328–34. doi: 10.1016/j.optom.2020.08.011, PMID: 34167928 PMC8569395

[32] Sohail M Palma-Martínez MJ Chong AY Quinto-Cortés CD Barberena-Jonas C Medina-Muñoz SG. Mexican biobank advances population and medical genomics of diverse ancestries. Nature. (2023) 622:775–83. doi: 10.1038/s41586-023-06560-0, PMID: 37821706 PMC10600006

[33] Hönekopp A Tommes LM Doebler P Weigelt S. Myopia prevalence, refractive status and uncorrected myopia among primary and secondary school students in Germany. Front Med. (2024) 11:1483069. doi: 10.3389/fmed.2024.1483069, PMID: 39726677 PMC11669525

[34] Wu PC Chen CT Lin KK Sun CC Kuo CN Huang HM. Myopia prevention and outdoor light intensity in a school-based cluster randomized trial. Ophthalmology. (2018) 125:1239–50. doi: 10.1016/j.ophtha.2017.12.011, PMID: 29371008

[35] Morgan IG French AN Ashby RS Guo X Ding X He M. The epidemics of myopia: aetiology and prevention. Prog Retin Eye Res. (2018) 62:134–49. doi: 10.1016/j.preteyeres.2017.09.004, PMID: 28951126

[36] Janssen I Medina C Pedroza A Barquera S. Screen time in mexican children: findings from the 2012 national health and nutrition survey (ENSANUT 2012). Salud Publica Mex. (2013) 55:484–91. doi: 10.21149/spm.v55i5.7248, PMID: 24626619

[37] Castagno VD Fassa AG Carret MLV Vilela MAP Meucci RD. Hyperopia: a meta-analysis of prevalence and a review of associated factors among school-aged children. BMC Ophthalmol. (2014) 14:163. doi: 10.1186/1471-2415-14-163, PMID: 25539893 PMC4391667

[38] Hyman L. Myopic and hyperopic refractive error in adults: an overview. Ophthalmic Epidemiol. (2007) 14:192–7. doi: 10.1080/09286580701535517, PMID: 17896297

[39] Lee KE Klein BEK Klein R Wong TY. Changes in refraction over 10 years in an adult population: the beaver dam eye study. Invest Ophthalmol Vis Sci. (2002) 43:2566–71. 12147586

[40] Mansfield A Addis M Mahalik J. Why won’t He go to the doctor?: the psychology of men’s help seeking. Int J Mens Health. (2003) 2:93–109. doi: 10.3149/jmh.0202.93

[41] Shen X Wang C Zhou X Zhou W Hornburg D Wu S. Nonlinear dynamics of multi-omics profiles during human aging. Nat Aging. (2024) 4:1619–34. doi: 10.1038/s43587-024-00692-2, PMID: 39143318 PMC11564093

[42] Fricke T Holden B Wilson D Schlenther G Naidoo K Resnikoff S. Global cost of correcting vision impairment from uncorrected refractive error. Bull World Health Organ. (2012) 90:728–38. doi: 10.2471/BLT.12.104034, PMID: 23109740 PMC3471057

[43] Mehta J Czanner G Harding S Newsham D Robinson J. Visual risk factors for falls in older adults: a case-control study. BMC Geriatr. (2022) 22:134. doi: 10.1186/s12877-022-02784-3, PMID: 35177024 PMC8855581

[44] Guo X Shakarchi AF Block SS Friedman DS Repka MX Collins ME. Noncycloplegic compared with Cycloplegic refraction in a Chicago school-aged population. Ophthalmology. (2022) 129:813–20. doi: 10.1016/j.ophtha.2022.02.027, PMID: 35245603

[45] Mimouni M Zoller L Horowitz J Wygnanski-Jaffe T Morad Y Mezer E. Cycloplegic autorefraction in young adults: is it mandatory? Graefe’s archive for. Clin Exp Ophthalmol. (2016) 254:581–5. doi: 10.1111/aos.12642 26686513

