# Supplementary material for: Prevalence and age-related trends of refractive errors in Mexican outpatients: a cross-sectional study
Source: Front Public Health. 2025 Oct 23;13:1675138. doi: 10.3389/fpubh.2025.1675138 (PMC12589056; doi:10.3389/fpubh.2025.1675138)
Supplement: Supplementary file 1 [file Supplementary_file_1.pptx]

## Slide 1
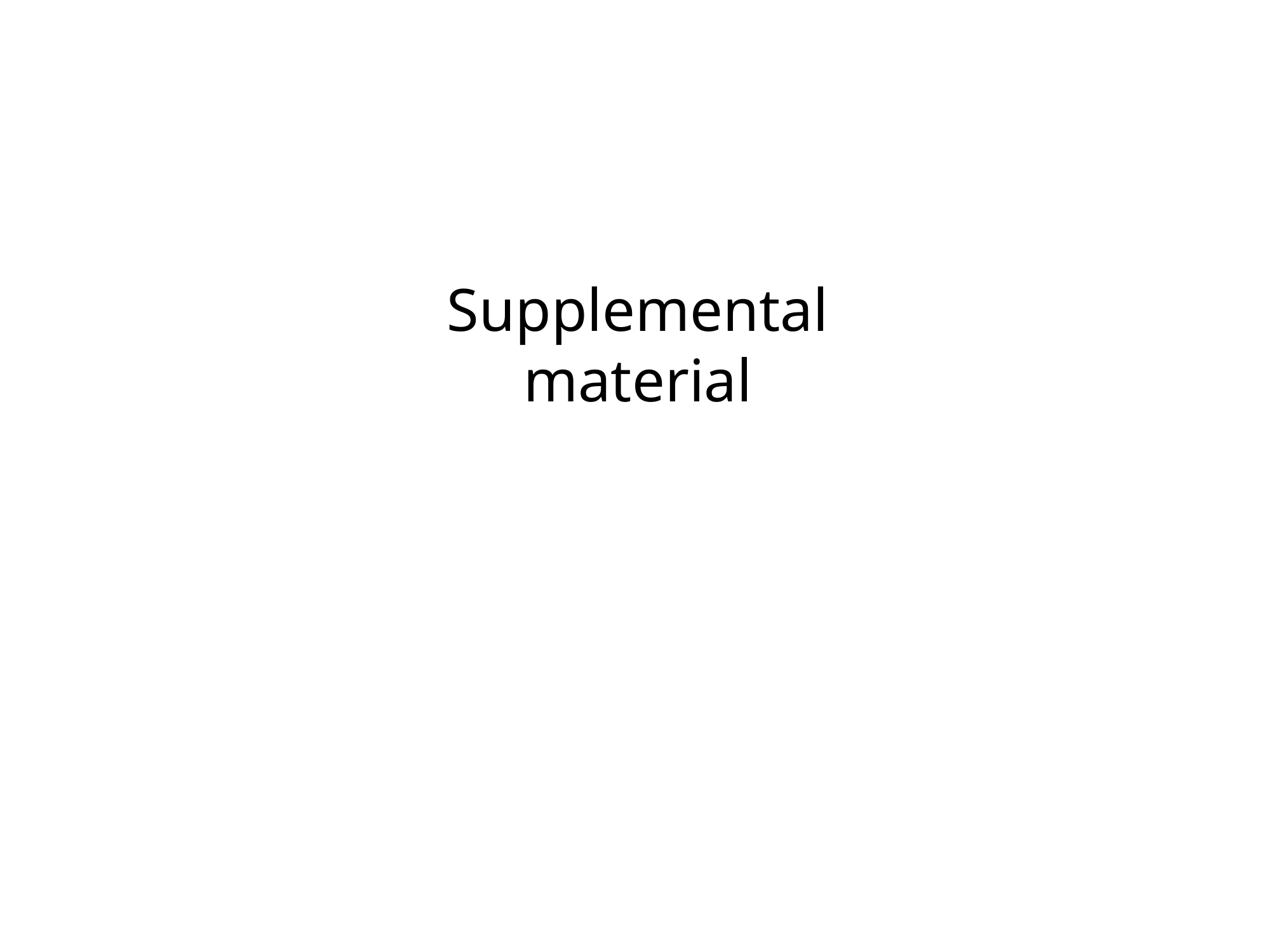

Supplemental material

## Slide 2
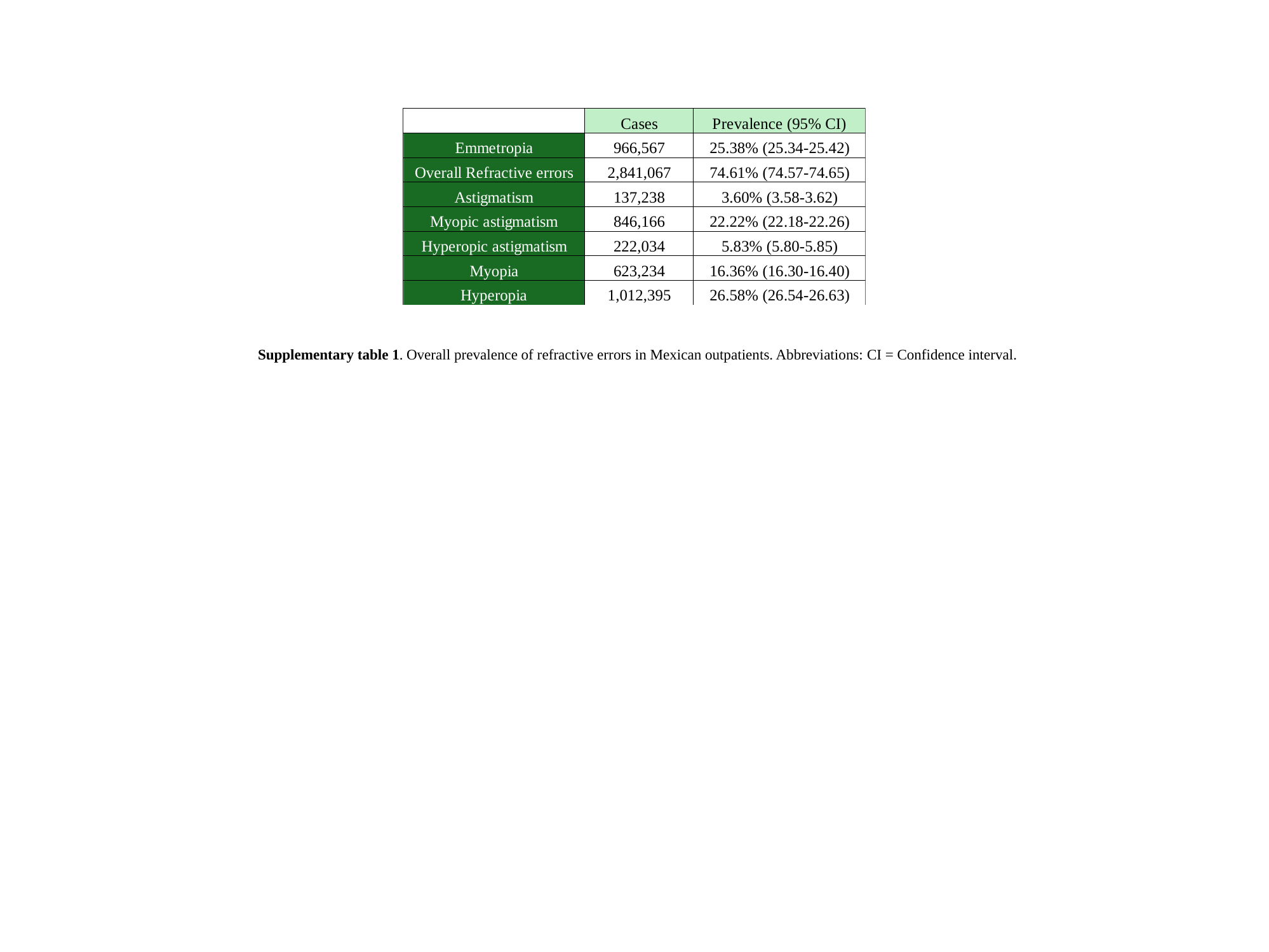

Supplementary table 1. Overall prevalence of refractive errors in Mexican outpatients. Abbreviations: CI = Confidence interval.

## Slide 3
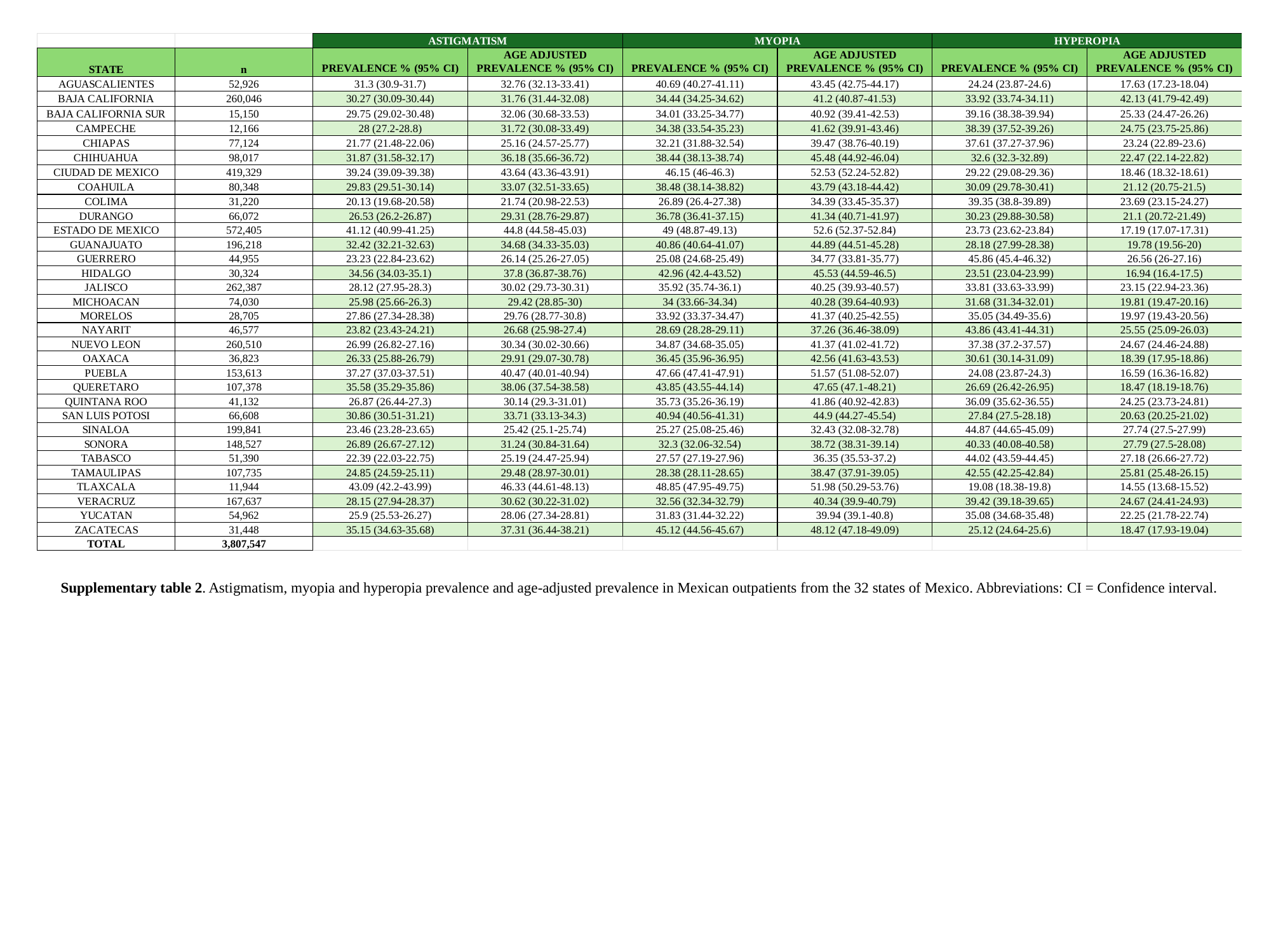

Supplementary table 2. Astigmatism, myopia and hyperopia prevalence and age-adjusted prevalence in Mexican outpatients from the 32 states of Mexico. Abbreviations: CI = Confidence interval.

## Slide 4
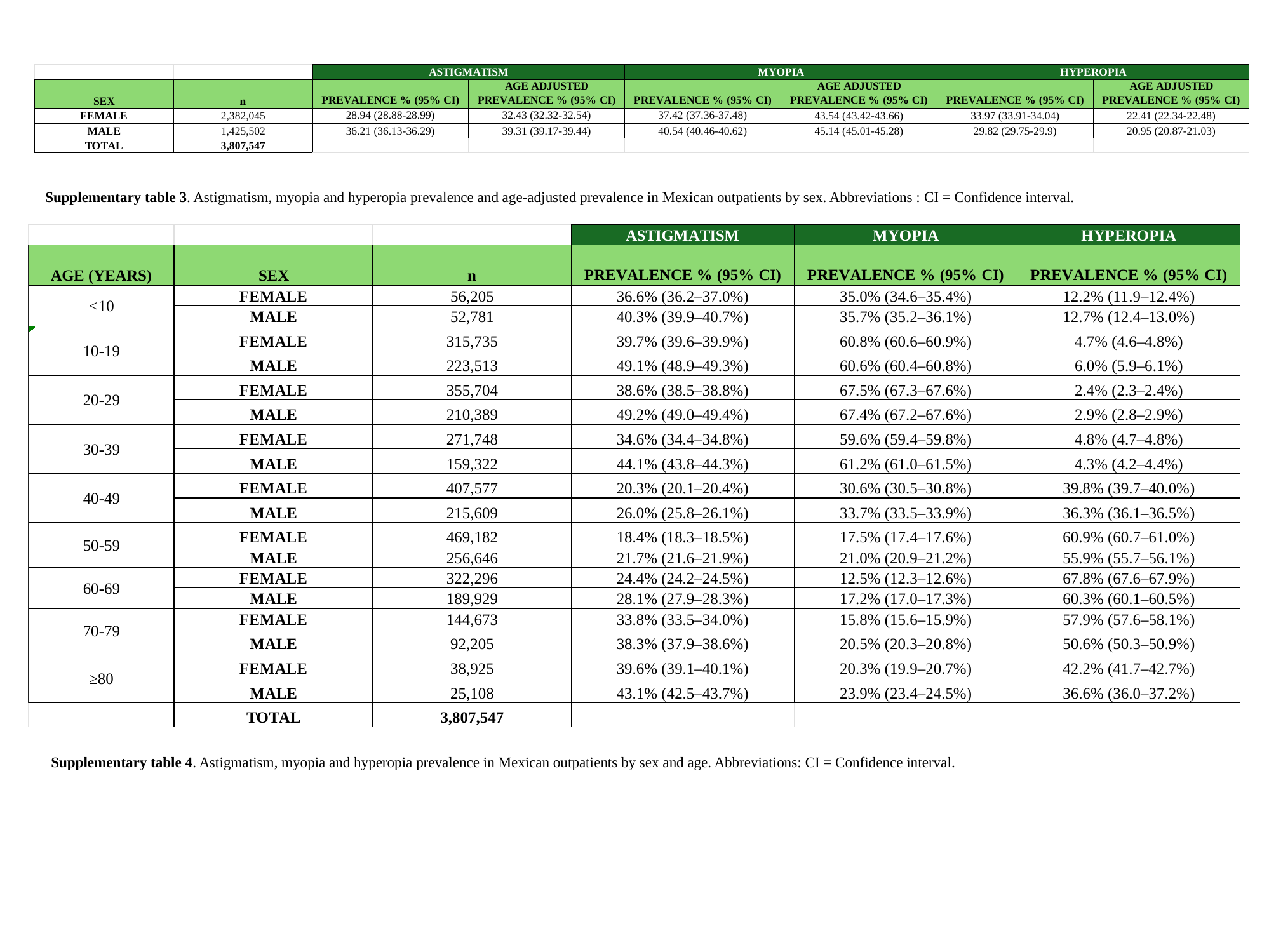

Supplementary table 3. Astigmatism, myopia and hyperopia prevalence and age-adjusted prevalence in Mexican outpatients by sex. Abbreviations : CI = Confidence interval.
Supplementary table 4. Astigmatism, myopia and hyperopia prevalence in Mexican outpatients by sex and age. Abbreviations: CI = Confidence interval.

## Slide 5
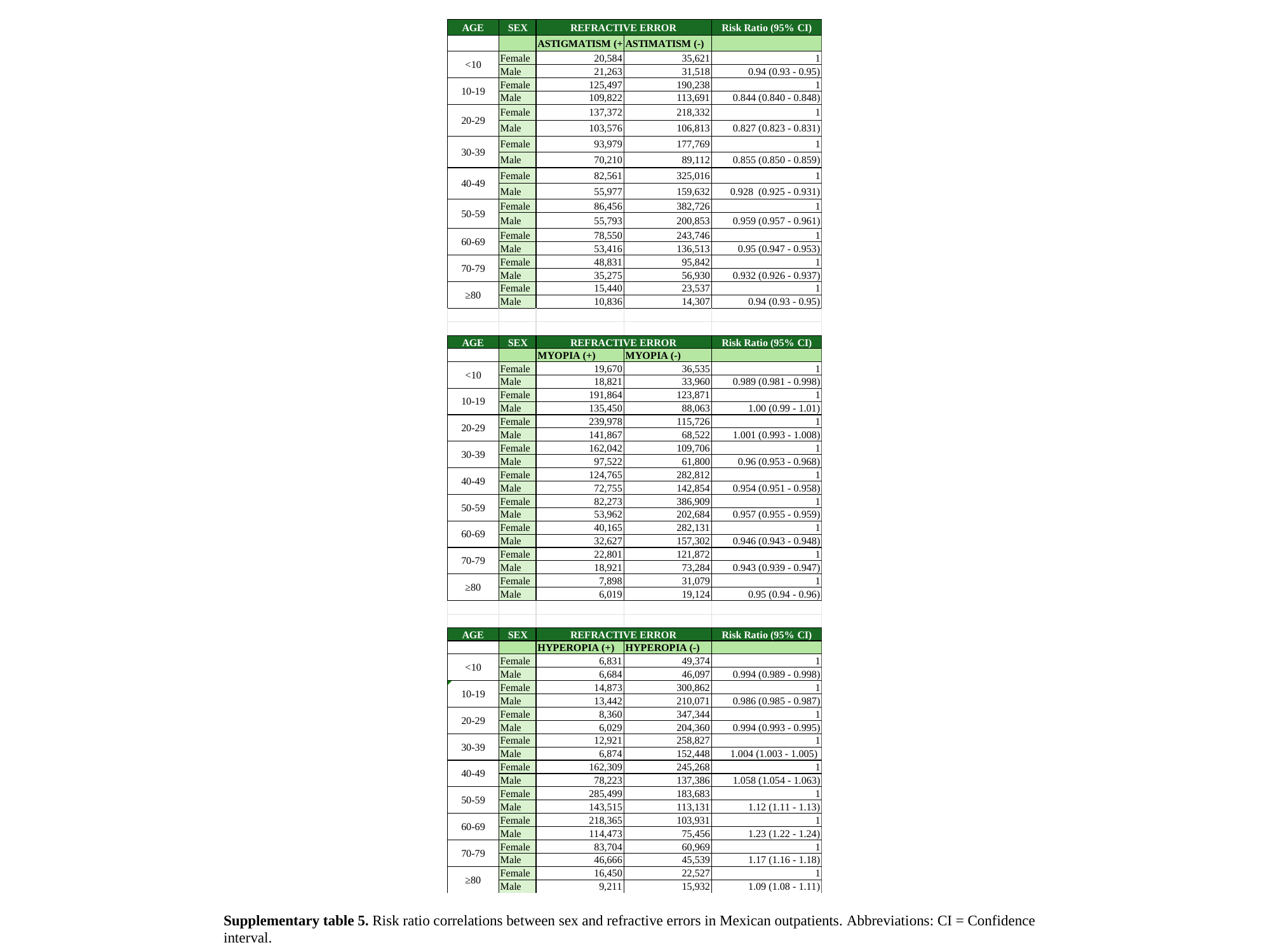

Supplementary table 5. Risk ratio correlations between sex and refractive errors in Mexican outpatients. Abbreviations: CI = Confidence interval.

## Slide 6
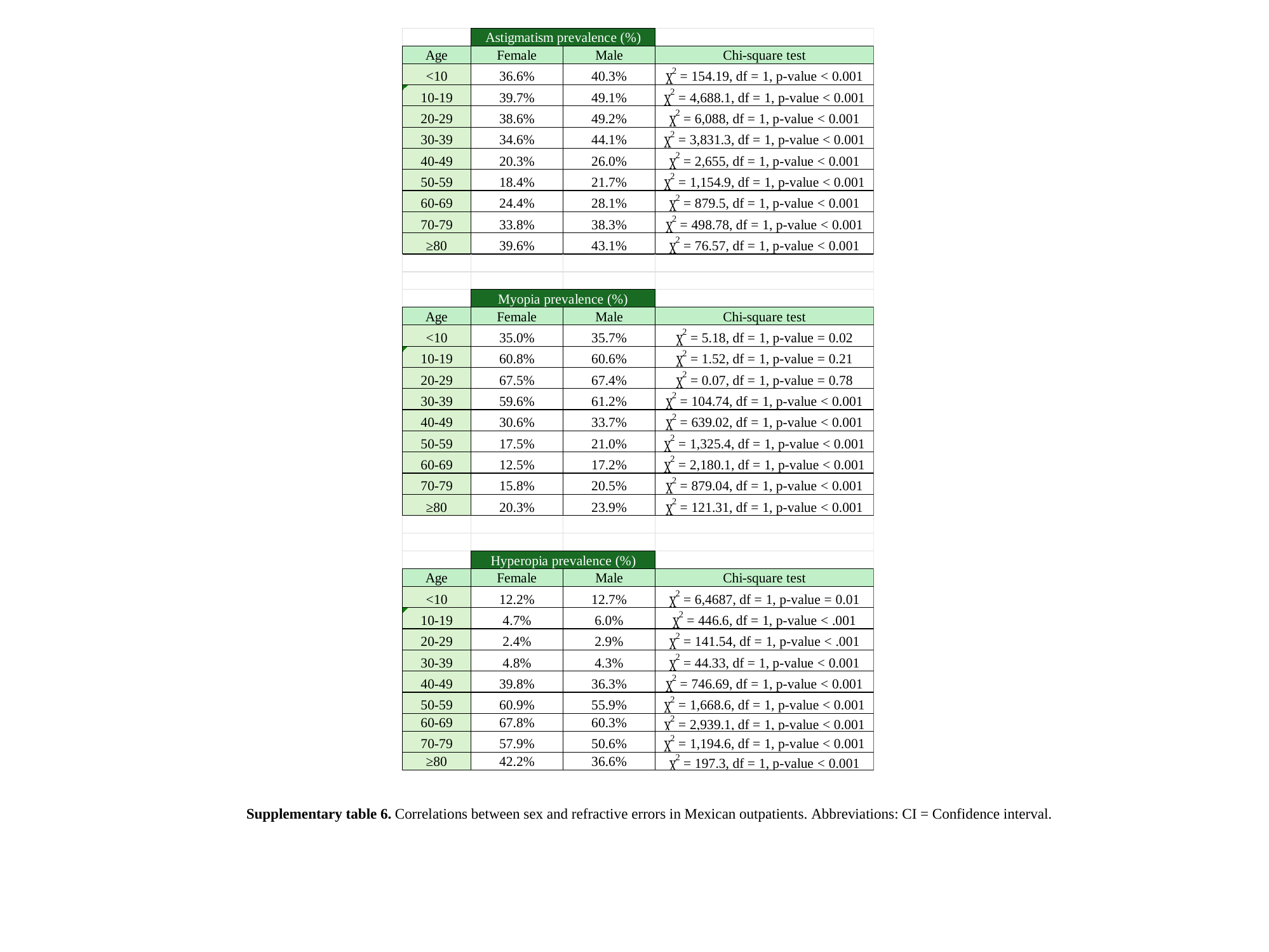

Supplementary table 6. Correlations between sex and refractive errors in Mexican outpatients. Abbreviations: CI = Confidence interval.
